# Deep Learning-Based Feature Silencing for Accurate Concrete Crack Detection

**DOI:** 10.3390/s20164403

**Published:** 2020-08-07

**Authors:** Umme Hafsa Billah, Hung Manh La, Alireza Tavakkoli

**Affiliations:** Department of Computer Science and Engineering, University of Nevada, Reno, NV 89557, USA; ubillah@nevada.unr.edu (U.H.B.); hla@unr.edu (H.M.L.)

**Keywords:** convolutional neural network, encoder-decoder architecture, semantic segmentation, feature silencing, crack detection

## Abstract

An autonomous concrete crack inspection system is necessary for preventing hazardous incidents arising from deteriorated concrete surfaces. In this paper, we present a concrete crack detection framework to aid the process of automated inspection. The proposed approach employs a deep convolutional neural network architecture for crack segmentation, while addressing the effect of gradient vanishing problem. A feature silencing module is incorporated in the proposed framework, capable of eliminating non-discriminative feature maps from the network to improve performance. Experimental results support the benefit of incorporating feature silencing within a convolutional neural network architecture for improving the network’s robustness, sensitivity, and specificity. An added benefit of the proposed architecture is its ability to accommodate for the trade-off between specificity (positive class detection accuracy) and sensitivity (negative class detection accuracy) with respect to the target application. Furthermore, the proposed framework achieves a high precision rate and processing time than the state-of-the-art crack detection architectures.

## 1. Introduction

Proper inspection and maintenance of civil infrastructures is essential to avoid costly and potentially life-threatening disasters. Manual inspection by civil engineers to assess structural defects employ heavy and large equipment and are time-consuming and labor-intensive. Furthermore, the manual assessment procedure could be potentially dangerous in inaccessible locations such as, under bridge decks and underwater beams. Autonomous civil infrastructure inspection systems are capable of continuously monitoring structural health with very little or no human intervention. Such autonomous robotic systems are designed to capture data for surface-level visual inspection and defect identification of civil infrastructures [1,2,3]. The main focus of this paper is on an autonomous inspection process for defect (crack) detection on concrete surface images.

Defective areas (i.e., cracks) on concrete surfaces exhibit two important properties. First, cracks do not have any definite shape or pattern. As a result, these defects do not represent any distinct feature properties, such as smooth curvatures or uniform width across their lengths. Second, cracks, when present, occupy a very small portion of the concrete surface. The rare occurrence and aberrant shape of these defects demonstrate the difficulty in utilizing generic pattern recognition approaches for detection and segmentation. Therefore, accurately detecting these anomalous appearances with statistical estimation techniques is a challenging task [4]. On the other hand, considering crack detection as an anomaly detection problem allows for devising suitable approaches for accurately and efficiently localizing such defects. Several anomaly detection techniques have shown promise from background subtraction [5,6] to unusual activity recognition [7].

### 1.1. Literature Review

Table 1 presents an overview of approaches for crack detection from the literature. As a general observation, there are three main approaches to addressing the problem of detecting cracks on concrete surfaces, i.e., methods based on image processing, techniques for pattern recognition and machine learning, and deep learning approaches. Image processing-based methods are quite simple, with the least amount of computational complexity and memory requirements, whereas deep learning-based techniques are the most computationally expensive, both in terms of memory requirement and operational cost. Despite computational efficiency of image processing techniques, these methods are generally not accurate or robust to concrete surface textures, exposure, and other geometrical features. Deep learning-based methods, on the other hand, have the highest accuracy, robustness, and generalization, expected from such computationally rigorous approaches. Techniques based on generic machine learning and pattern recognition approaches are share the low cost and computational efficiency of the image processing-based techniques, and modest accuracy and robustness of techniques based on deep learning. However, to achieve the full potential of machine learning-based techniques, suitable feature engineering is required. This could be problematic, since concrete cracks generally do not possess any distinguishing geometric or photometric representations. Below, we will discuss each of these techniques in more detail.

**Figure 1:**
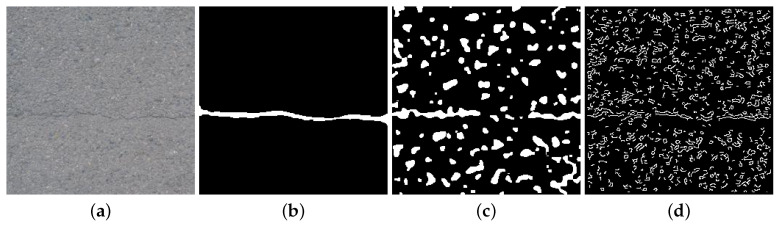
Challenges in crack detection using image processing techniques: Although cracks are edge-like features, they usually represent defects not associated with other edges. (**a**) Original image, (**b**) cracked pixels ground truth, (**c**) sharp transition changes using Difference of Gaussians (DoG), (**d**) Canny edge detection. As it can be seen, edge detection produces other sharp color/contrast transitions along with crack pixels.

**Approaches Based on Image Processing:** A number of techniques for crack detection based on image processing have been investigated. These include thresholding [8,9,10,11,12], morphological operations [13,14,15,16,17,18] and edge detection algorithms [19,20,21,22]. Although image processing techniques are computationally inexpensive, they have several disadvantages. These techniques generate unnecessary feature points on highly textured concrete images. Removal of these feature points (using median or mean filtering) could also result in removing portions of cracked regions. As a result, traditional image processing techniques fail as robust crack classification methods [3,20,23]. Moreover, concrete defect images are affected by environmental non-uniformity such as illumination, noise, and shading, to which image processing methods are highly sensitive [3,23]. Therefore, image processing methods such as Difference of Gaussians (DoG) and Canny edge detection lack sufficient robustness to be effectively employed as crack detectors. Figure 1a shows an concrete image with a relatively clear crack across the image, seen in Figure 1b. Results of DoG and Canny algorithms are shown in Figure 1c,d and showcase the inability to distinguish between sharp changes in color and intensity of the image from real crack pixels.

**Approaches Based on Machine Learning:** Machine learning architectures, including Support Vector Machines (SVM) [13,21,24], Adaboost [21], and Multi-Layer Perceptron (MLP) [25] networks, have also been employed to improve robustness of crack identification. More recently, a combination of machine learning and image processing techniques were employed to improve the defect detection results [26,27]. Although using elaborate machine learning approaches helps with increasing the accuracy of defect detection, these techniques are computationally expensive and require appropriate parameter selection. In addition, these techniques require effective preprocessing of the input images, engineering suitable feature spaces capable of discriminating crack features from other normal pixel intensity differences or textures, and effective training [4].

**Deep Learning Classification Approaches:** The process of backpropagation allows for effective updating of weight parameters in artificial neural networks (ANN). Therefore, using multiple hidden layers and a robust optimization algorithm allows these networks to learn specific discriminating features that distinguish crack locations from other areas on the surface [28,29]. However, in order to effectively encode suitable discriminating features, a sufficiently deep network needs to be utilized. Convolutional neural networks (CNNs) allow for efficient implementation of deep neural architectures [30]. CNN architectures closely simulate the functionality of the biological visual cortex by representing cortical areas as individual layers. These layers extract unique feature sets for a specific dataset and learn their statistical properties.

The astounding performance of these networks in many image classification and object recognition applications has inspired researchers to investigate their use for concrete defect identification [3,23,31]. Classification-based CNN architectures for concrete crack identification divide an image into multiple sub-blocks and classify each sub-block as a region containing cracks or regular surface regions. Among traditional CNN architectures employed for concrete crack identification applications are ResNet [32,33,34], AlexNet [35,36], and VGG-Net [37]. Recently, custom CNN architectures have been designed specifically for concrete crack identification purposes [3,23,31,38]. The main disadvantage of these methods is that they consider crack identification as an image classification problem. Although these techniques are highly efficient for crack feature extraction, they need to utilize image processing algorithms as a post-process to effectively identify the exact location of crack [3,23].

**Deep Learning Segmentation Approaches:** Semantic segmentation addresses the problem of localizing and classifying concrete crack pixels at the same time. There exist several semantic segmentation architectures for scene parsing, object instance segmentation, and many other applications. Among them, FCN [39], Mask-RCNN [40], UNet [41], and SegNet [42] have achieved significant performance benchmarks. These architectures learn important feature attributes of regions within an image through a series of encoding operations and project back the encoded information onto the image space through decoding operations. The decoded projection back onto the image space can be essentially used as different region labels, thus resulting in the segmentation of the input image into its constituent subregions. These architectures perform a series of encoding and decoding operations for segmentation. Among semantic segmentation deep architectures, SegNet architecture outperforms UNet and FCN in terms of accuracy, computational complexity, and memory usage.

One of the main difficulties in designing very deep architectures lies in the gradient vanishing problem, by which the performance of deep networks plateaus due to insufficient gradient values to backpropaget through the network. The ResNet [35] architecture allows for propagating residual values via the forward pass of the network, thereby addressing the vanishing gradient problem. Inspired by this, ResNet-like architectures can be employed in an encoder/decoder manner for segmentation purposes. However, these frameworks involve a huge number of parameters.

In recent years, a number of encoder/decoder architectures have been proposed with applications to crack detection [4,43,44,45,46,47,48]. These encoder/decoder-based CNN architectures (SegNet [42], UNet [41], FCN [39]) first encode a suitable feature space capable of discriminating crack-like features into a lower dimensional manifold through a series of encoders. This feature space is then decoded back into the higher dimensional image space through corresponding decoders. Salient feature attributes of each pixel within the input dataset are learned through these encoding and decoding operations, associating each pixel with a class label.

There are certain drawbacks to encoder/decoder architectures (both for crack detection and semantic segmentation) that negatively impact their performance. First, the computations performed by these architectures are twice those of regular CNN classification architecture [30]. This will have a negative impact on the network’s sensitivity to gradient vanishing problem as well as environmental non-uniformity. Moreover, effective training of these deep networks require a balanced dataset of crack-like pixels and regular pixels. As mentioned earlier, in a given image, crack pixels constitute a very small portion of the surface. Extracting discernible feature sets from these highly imbalanced crack/non-crack data becomes an impeding challenge for traditional segmentation networks.

### 1.2. Contributions

The main contributions of the proposed architecture are represented as follows:

- In this paper, we propose an efficient framework for class imbalanced datasets such as crack detection. The proposed framework incorporate an encoder-decoder network architecture for reducing the effect of gradient vanishing problem. The network architecture (referred to as ANet-FSM) is discussed in Section 2. Additionally, our investigation reveals that using this specific type of architecture is effective in applications suffering from high degrees of class imbalance–crack identification (non-crack pixels are much more than crack pixels). Empirical evidence supporting the propositions in this work are represented in Section 2.
- The second main contribution of this study is the incorporation of a feature silencing module (FSM). The FSM module resolves the sensitivity of the deep architectures toward feature maps that do not contribute effectively to the optimization of the network loss. The incorporation of the FSM with the proposed CNN architecture significantly reduced the false classification rate of the concrete crack detection process. We have explained the functionality of the FSM in Section 2. The efficiency of the FSM on concrete crack identification dataset is represented in Section 3.

## 2. Methodology

In this section, we explain the modules of our proposed architecture. To represent our network architecture comprehensively we briefly discuss the functionality of a CNN and encoder-decoder-based architecture in the following sections. Later, different modules of the proposed architecture are explained.

### 2.1. Data Acquisition

The visual data were collected by the robot developed by our team [2], which is programmed to stop at every 2 feet (approximately 60 cm) and take an image of the bridge deck. Therefore, we only have visual data under static condition (not in video capture mode also referred to as dynamic data acquisition) in which crack detection is performed.

### 2.2. An Encoder-Decoder Architecture

**Figure 2:**
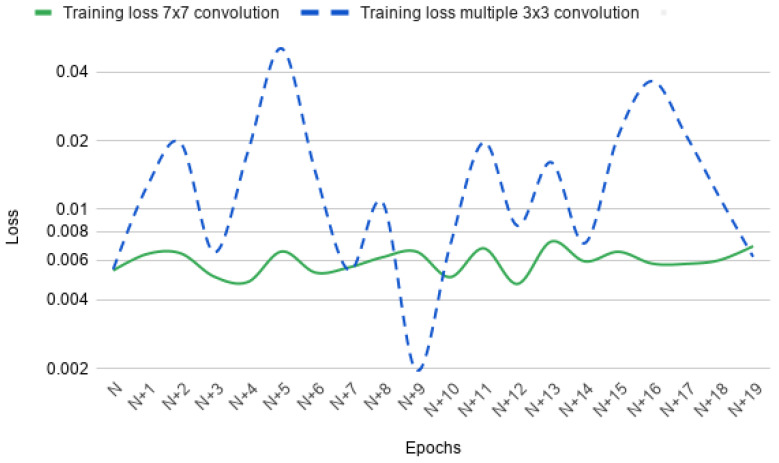
Training loss of two different type convolution networks. The training loss was extracted from a 20 epoch window while training. N depicts the start of the window and the loss was plotted for each epoch. Training loss of convolution network with two different kernel sizes (3×3 and 7×7) are plotted.

For crack identification purposes, a number of different encoder-decoder-based architectures were proposed recently such as InspectionNet [43], DeepCrack [44], SDDNet [45], and SegNet-SO [4]. Although these architectures represent promising results on crack classification, they have some drawbacks. Firstly, these architectures extract crack feature attributes using multiple 3×3 convolution operation. Multiple convolution operations of small receptive field extract both global and local feature attributes of the input image as reported in [37]. While going through multiple convolution operations in deeper layers, the gradient stability of a network is lost. The network stops updating the weights of these parameters, which results in the wrong classification of crack pixels. As a result, the network attains a very high loss value. An example of this phenomenon is represented in Figure 2. In this figure, the loss of two network architectures involving two different convolution operations (3×3 and 7×7) is plotted. We have extracted a 20 epoch window from training for better visualization of the loss. It is evident from the figure that, for multiple 3×3 convolution operations, the loss is not stable between any two epochs. The loss doesn’t change gradually for this convolution operation. For example, the loss in epoch N+1 jumps to a higher loss in comparison to the loss in epoch *N*. This phenomenon is visible through the entire 20 epoch window and represents the high gradient instability of this network. On the other hand, for the network with 7×7 kernel size, the loss changes gradually. This represents the stable gradients of the network.

The loss value of both the networks in Figure 2 lie within μ±σ, where μ is the mean value of the loss in current epoch window and σ is the standard deviation. For the network using 3×3 convolution kernel, the loss lies within 0.002±0.012, where μ=0.02 and σ=0.012. The maximum and minimum loss value of this network is 0.05 and 0.002, respectively. On the other hand, for the network with 7×7 convolution kernel, the loss lies within 0.006±0.0007, where μ=0.0006 and σ=0.0007. The maximum and minimum loss of this network are 0.0072 and 0.005 respectively. These statistics represent that the loss generalization of a single large convolution kernel (e.g., 7×7) is better than multiple small kernels (e.g., multiple 3×3 kernels). The loss value of the network with multiple small convolution kernels is seven times (approximately) higher than the network with a large convolution kernel. Due to gradient instability, the network did not update many parameter weights, resulting in a very high loss value of 0.05. Furthermore, the high standard deviation also represents the highly unstable gradient values or the gradient vanishing problem.

**Figure 3:**
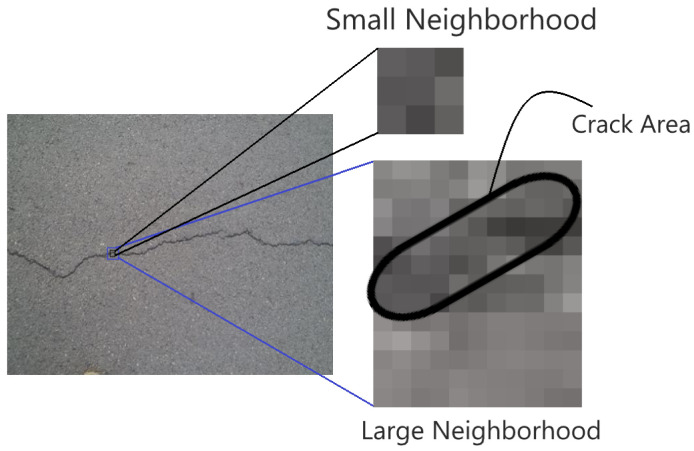
The comparison of two neighborhood of crack location from a small neighborhood and large neighborhood. Since crack pixels occur a very small amount of time, the small neighborhood only contains crack pixels. On the other hand, in the large neighborhood, the statistical relationship between crack and non-crack pixels can be captured more appropriately.

Secondly, the concrete defect identification datasets vary significantly from state-of-the-art image classification databases because crack pixels show anomalous behavior (aberrant patterns and shapes) in comparison to healthy concrete pixels. Additionally, these anomalous pixels appear only in a small portion (2–10% approximately) of a concrete image (known as class imbalance problem). Therefore, the relationship between crack and non-crack pixels can be extracted more efficiently if the convolution operation is performed concerning a large neighborhood. An example of this phenomenon is represented in Figure 3. The image in this figure is extracted from the Crack260 [44] dataset. The enlarged neighborhood of a crack location is shown in this Figure 3. It is evident that, with the small spatial neighborhood, we can extract the properties of crack only. Although the global and local features can be extracted using multiple spatial neighborhoods, it is crucial to extract the anomalous relationship between crack and non-crack pixels. This relationship can be extracted using a relatively large neighborhood [45].

For concrete crack classification, the deep crack [44] network architecture achieved a significant performance using multiple receptive fields of size 3×3. This approach has the advantage of extracting efficient local and global features of crack pixels. As discussed earlier in Figure 2, the gradient vanishing problem is very prominent in these types of approaches. To address this issue another deep architecture was developed for crack detection namely SDDNet [45]. This architecture uses a combination of different convolution operations such as separable, atrous, and point-wise convolutions. They used a large receptive field for extracting the crack and non-crack pixel relationship. In this paper, we proposed a crack detection framework with only one type of convolution operation. We used a 7×7 convolution kernel with only one convolution layer in each encoding operation. The large receptive field is very crucial for extracting the imbalanced relationship between crack and non-crack pixels as shown in Figure 3.

### 2.3. ANet-FSM Architecture

**Figure 4:**
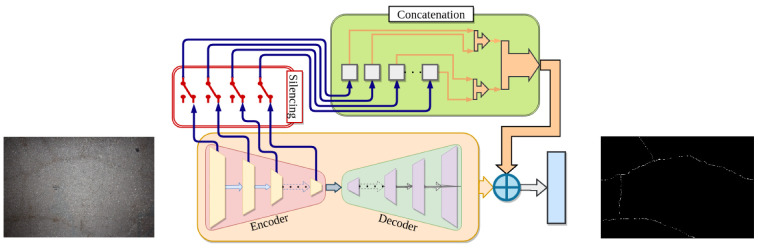
The proposed network architecture overview.

**Figure 5:**
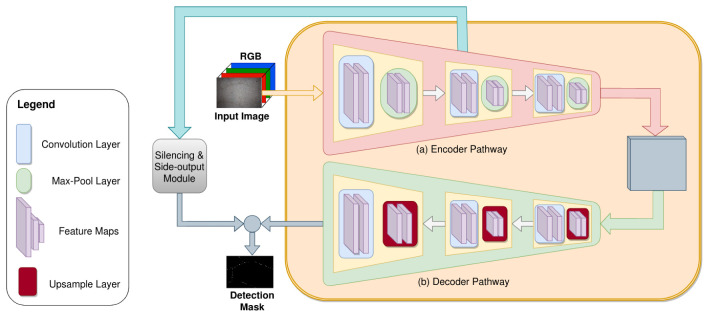
Encoder and decoder module of Attention Network (ANet)-feature silencing module (FSM) architecture. Each encoder performs a 7×7 convolution and max-pooling operation. The decoders up-sample this low dimensional feature space into upper dimension using bi-linear interpolation. The feature space decoding is performed by the convolution operation in each decoder.

The proposed ANet-FSM architecture is comprised of an encoder-decoder module, a feature silencing module (FSM), and a concatenation module. An overview of the whole architecture is shown in Figure 4. Important crack feature attributes are learned through the encoding and decoding operations. The FSM eliminates weak feature maps generated from the encoder module and passes the strong feature maps to the concatenation module. These feature maps are up-sampled and concatenated together in the concatenation module. The up-sampled feature maps along with the output feature map of the decoder module are passed to a 1×1 convolution and soft-max layer. The encoder module in Figure 5 is composed of five encoder layers.

Each encoder layer assembles a convolutional layer and a max-pooling layer. Two auxiliary layers (Batch Normalization and ReLU) are added at the end of each convolutional layer. The convolution layers in each encoder use 4,8,16,32, and 64 kernels of size 7×7, respectively. The max-pooling operation down-samples the feature space using a 2×2 pooling window. Additionally, this operation saves the pooling indices in memory to be used in the up-sampling operation in the decoder module.

A representation of the decoder layers for each corresponding encoder is represented in Figure 5. Each decoder layer performs a bi-linear up-sampling operation on its input feature space using the pooling indices saved during max-pooling operation. A convolutional layer is employed at the end of each up-sampling operation to decode the feature space of its corresponding encoder.

A spatial neighborhood of size 7×7 is used for each convolution operation throughout the encoding and decoding operation. As discussed earlier in Section 2.2, this neighborhood is also efficient for eliminating the gradient vanishing problem of state-of-the-art crack detection and semantic segmentation architectures. Therefore, each encoder layer in this architecture is comprised of only one convolutional layer.

On the other hand, the max-pooling operation eliminates some feature responses in the course of generating position invariant feature space. This feature loss is nominal when enough instances of different classes are present. The concrete defect datasets are highly imbalanced due to the low occurrence of defected pixels. Removal of these feature responses significantly affects the performance of the network. A transient solution to this problem is to up-sample the feature spaces into the original dimension after each encoding operation [4].

**Figure 6:**
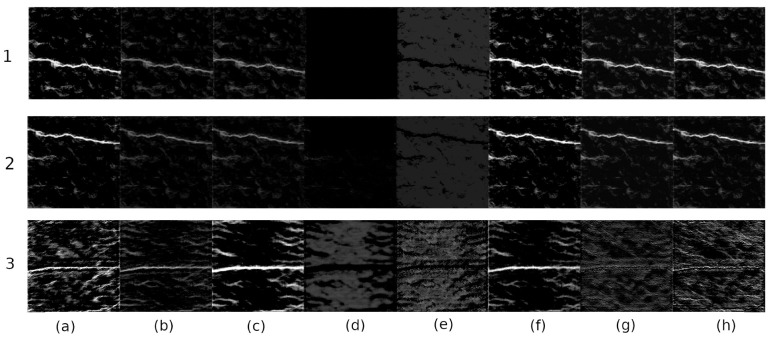
An example of a feature space of different crack images (rows) during an encoding operation. Some feature responses are strongly indicative of crack presence (**a**–**c**,**f**,**h**), while weak feature responses (**d**,**e**,**g**) generate a weight matrix close to zero. The weak feature maps are eliminated using Equation (1).

**Figure 7:**
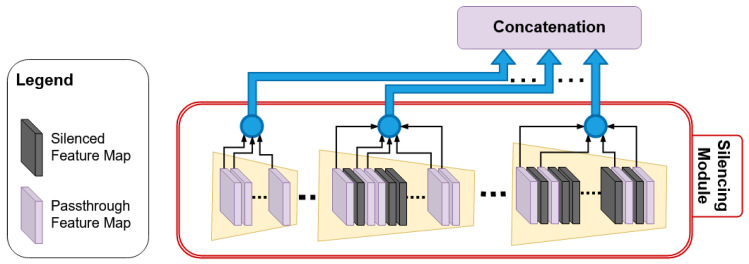
FSM of the proposed architecture: the silenced feature maps are represented with black colors.

Empirical analysis performed on the feature spaces after each encoding operation reveals that all the features in the feature space do not contribute equally to the crack pixel identification. An example of a feature space extracted after a single encoding operation is shown in Figure 6. It is evident from the figure, the feature maps in Figure 6(1–3)a–c,f,h represent very strong crack features. These feature maps contribute significantly to final crack pixel identification. The remaining feature maps in Figure 6(1–2)d,e, and (3)g are responsible for overlapping the salience property of the feature space Moreover, the weight matrix of these feature space contains gradient values close to zero, which results in false classification. To enhance the precision of crack classification, the FSM module in ANet-FSM architecture eliminates these feature maps from the network feature space. A graphical representation of the FSM module is shown in Figure 7.

The FSM module in Figure 7 uses Equation (1) for extracting significantly contributing feature maps from each encoder feature space. Equation (1) is defined as follows:

(1) $$\begin{array}{c} F_{p}=\overset{i=1\to N}{\underset{f_{i}\in F}{⋃}}f_{i}\leq th\\ F_{s}=F\setminus F_{p} \end{array}$$

where $F_{p}$ is the pruned feature space, *F* is a feature space from an encoder, $f_{i}$ is the *i*th feature map in *F*, th is a threshold value, $F_{s}$ is the selected features for passing to the concatenation module, and *N* is the number of feature maps in feature space *F*.

The encoding operation generates some significantly contributing feature maps for crack detection. Using Equation (1), we extract these feature maps for up-sampling. The rest of the feature maps are considered weak by this equation. As a result of gradient vanishing, these feature maps have values close to zero. Hence, in Equation (1), we select the feature maps for pruning ($F_{p}$) having values less than a threshold th. A new feature space $F_{s}$ is formed by eliminating the feature spaces in $F_{p}$ from the original feature map *F*.

The decoder layer in ANet-FSM uses the pooling indices from the max-pooling operation of the corresponding encoder for performing up-sampling (introduced by [42]). The decoding operations are directly dependent on the feature space of the corresponding encoders. Propagating the pruned feature space from one encoder layer to another encoder layer effects the pixel connectivity of the cracks as well as the precision of the network. The feature spaces in encoder and decoder propagate without any pruning operation. Additionally, the pruned feature space ($F_{s}$) is passed to the concatenation module shown in Figure 4. This concatenation module performs bi-linear up-sampling on the pruned feature space from each encoder (except the first encoder). These up-sampled pruned feature spaces are concatenated with the feature space of the final decoder. Since the crack classification is a one-class classification problem, we use a 1×1 convolution operation at the end of the concatenation operation. This convolution operation helps to estimate the crack location from the concatenated feature space.

An example of eliminated feature maps from an instance of the Illinois Bridge dataset is represented in Table 2. To represent the effectiveness of the FSM module in reducing the complexity of a network, we have introduced three measures such as feature silencing rate (FSR), feature space size before pruning ($FSS_{b}$), and feature space size after pruning $FSS_{a}$. Since there is a scarcity of standardization metrics of feature space pruning, we have incorporated the above three measures for evaluation only. The FSR in Table 2 is defined in Equation (2).

(2) $$FSR=\frac{B_{s}-A_{s}}{B_{s}}\;$$

where $B_{s}$ represents the number of features before silencing and $A_{s}$ represents the number of features after silencing. Additionally, we have analyzed the feature space size before and after silencing using Equation (3).

(3) $$\begin{array}{c} FSS_{b}=x\times y\times B_{s}\\ FSS_{a}=x\times y\times A_{s} \end{array}$$

where *x* is height of an image, *y* is width of an image, $FSS_{b}$ is the the feature space size before silencing, and $FSS_{a}$ is the feature space size after silencing. In Table 2, we have calculated the value of Equation (3) for a 256×256 image.

Considering the low number of feature maps generated by the first layer encoder, the FSM module does not eliminate feature maps from the feature space generated by this encoder. The feature space from the second and third layers are reduced to half of their original size. In the fourth layer more than half of the feature maps are eliminated from feature space. In total, the FSM eliminates 51% of the feature maps generated from the encoder module. Additionally, feature space size value is significantly reduced after silencing. This represents the effectiveness of the FSM module in reducing the cost of the network. It is important to note that, by observing the network parameters, almost half of the computations in this encoder-decoder-based architecture, do not contribute significantly to the prediction. Elimination of such features improve the performance as well as reduces the computations of the network.

## 3. Experiment Results

In this section, we discuss the results of different deep architectures on concrete crack identification. The dataset preparation and augmentation methods are described first. Then we represent our experimental setup and result in analysis in the following sections.

### 3.1. Dataset Preparation

The scarcity of a well-balanced dataset is a challenge for anomalous pixel identification applications such as concrete crack classification. Since the occurrence of crack pixels is much lower than healthy concrete pixels, it is essential to train a deep network architecture with an enormous amount of data instances containing crack pixels. Therefore, we have collected concrete images of different highway bridge decks from different parts of the USA using our previously developed nondestructive evaluation robots [2,3,49] at the Advanced Robotics and Automation Laboratory. We captured images in various light illumination and different times of day and night to incorporate the non-uniformity of the environment as much as possible in our dataset (referred to as Illinois Bridge dataset).

**Figure 8:**
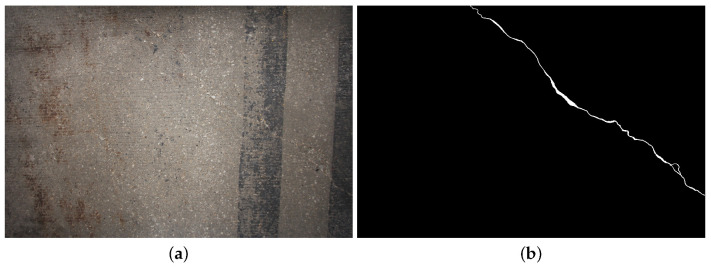
(**a**) Sample crack image and (**b**) annotated image from the Illinois Bridge dataset.

Appropriate annotation of the images in the dataset is very important for training and validating CNNs. This annotation process is arduous due to the difficulty involved in finding the crack pixels in an image (considering their low occurrence). As a result, the pixels were color-coded for each image individually from the Illinois Bridge dataset. The crack pixels were assigned a white color and the healthy concrete pixels were assigned a black color. An example of our annotated image dataset is shown in Figure 8.

The annotated Illinois dataset consists of forty-six concrete crack images and their corresponding binary labels. Each image in the dataset has a resolution of size 5000×3000. We divided the original dataset into two separate datasets such as training and testing. The training dataset includes 80% of the original dataset and the validation dataset includes the rest of the dataset. Additionally, a testing dataset was prepared to consist of 200 imaged of size 1024×1024 collected from different bridge decks.

There exists a number of published datasets such as Crack260 [44], CrackForest [50]. These datasets consist of very low resolution images (256×256, 512×512) in comparison to Illinois Bridge dataset. Additionally, the Illinois Bridge dataset consists of images with several types of noise such as vegetation, road paint, oil spilling and many more. An example of the annotated dataset is shown in Figure 8a.

The crack inspection dataset represents high-class imbalance property. To extract appropriate crack features, it is essential to provide the network with enough instances of crack samples. Therefore, we employed a data augmentation technique proposed by [4] for this purpose on our original dataset.

**Figure 9:**
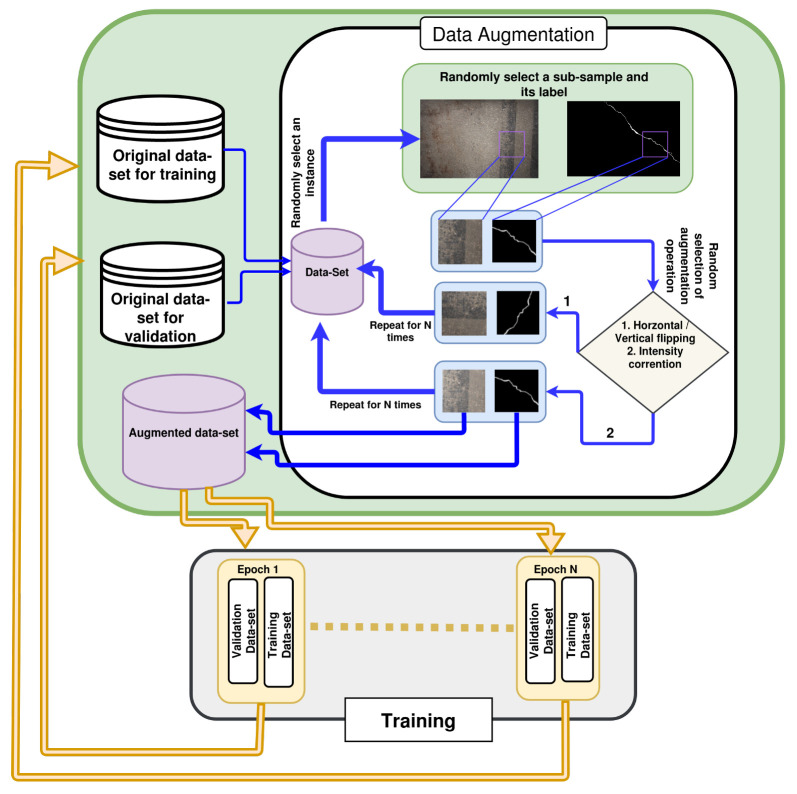
The training procedure with data augmentation technique.

The data augmentation technique randomly selects an image from the original dataset (training or validation). A random sub-sample of the selected image is chosen for the data-augmentation operation. This operation is chosen randomly for each individual sub-samples. The data-augmentation operations used in this work are horizontal flipping, vertical flipping, and gamma correction of intensity. The gamma value is also chosen randomly for intensity correction. This process is repeated for *N* times, where *N* represents the size of the dataset. As a result, this technique can generate *N* sub-samples of augmented dataset from the original high resolution dataset. A graphical representation of the data augmentation technique is shown in Figure 9.

### 3.2. Experimental Setup and Result Analysis

The proposed network architecture was trained on a 1080 Gtx GPU with 10 Gb memory. For hyper-parameter optimization, Adam optimizer was used with a learning rate of 0.0001.

The proposed deep network architecture was pre-trained on the Illinois Bridge dataset for 300 epochs. Many state-of-the-art CNN network training involves transfer learning from existing networks such as VGG-16 [37]. This transfer learning from the existing network has several disadvantages in our case. Firstly, transfer learning from state-of-the-art image processing datasets (ImageNet) will give the network unnecessary information on various objects. Since crack pixels have a very small amount of occurrence in an image, the transfer learning process may overlap the features of crack. Secondly, the ANet-FSM architecture prunes feature space weights below a threshold (Equation (1)). Transferring weights from another module (or network) initializes the feature space with high weights. As a result, the FSM module cannot find the weak feature maps for pruning. For these reasons, the network was pre-trained on the Illinois Bridge dataset instead of performing transfer learning.

The ANet-FSM network was trained for 300 epochs. At each epoch, datasets for training and validation are generated using the previously discussed data augmentation technique from the respective original training and validation datasets. In Figure 9, we represented the process of data-augmentation and training visually. Each image of the training and validation dataset generated by the data-augmentation technique has a resolution of 512×512. The training dataset consists of 5000 images and the validation dataset consists of 1000 images. As a result, a dataset of 6000 images is generated in each epoch in training. In total, the network is trained and validated on 300 different datasets, respectively.

State-of-the-art data augmentation techniques generate an augmented dataset from a handful of low-resolution images for crack detection. As result, training is performed on the same dataset for each epoch. This type of training procedure is highly susceptible to an overfitting problem. Nonetheless, the data-augmentation technique and training process incorporated in this paper generates different training and validation datasets for each epoch at the time of training. As a result, the network sees a range of different types of images. The overfitting problem of the traditional training procedure is overcome through this training procedure.

We evaluate the performance of the pre-trained network using the test dataset of 200 images. The testing image size (1024×1024) was selected to be larger than the training image size (512×512) to represent the robustness of the network towards noise in large resolution images. Moreover, the computational complexity involved with large images generates the gradient vanishing problem in a CNN architecture. The effect of the gradient vanishing problem is more evident in large scale images. Therefore, the resolution of test dataset images is twice as large as the train dataset.

The performance of ANet-FSM architecture was compared with two different types of encoder-decoder architectures such as semantic segmentation and crack detection architecture. The semantic segmentation architectures such as SegNet suffer from the gradient vanishing problem as discussed in Section 1. The effect of this problem is strongly evident in class-imbalanced datasets (crack detection dataset). This effect was evaluated by comparing with semantic segmentation architecture (SegNet [42]). Moreover, several crack segmentation architectures were employed for performance comparison such as InspectionNet [43], SegNet-SO [4], Deep crack [44], and SDDNet [45].

The proposed network architecture was evaluated based on three different criteria such as network complexity, qualitative measurement, and qualitative analysis. The complexity of different networks was analyzed in terms of the number of major computations performed. Later, a quantitative and qualitative analysis was performed on the results of different deep network architectures.

#### 3.2.1. Network Complexity Analysis

One of the solutions for tackling the gradient vanishing problem of very deep networks is the reduction of computational complexity. Since the convolutional layers are responsible for salient feature attribute extraction in CNN architecture [51], most of the computations are performed by this layer. As a result, the complexity of the network was measured based on the computations associated with the convolutional layers. The network complexity is defined as $K_{s}$ in Equation (4).

(4) $$C=\sum_{i=0}^{E+D}\left(N_{C_{i}}\times K_{s}\right)\times N_{k_{i}}$$

where $N_{C}$ is the number of convolution layers, $N_{k}$ is the number of kernels in each layer, $K_{s}$ is a m×n dimensional kernel (*m* is height and *n* is width), *E* is the total number of encoders, and *D* is the total number of decoders. Since the network architectures discussed in this work use five encoders and five decoders, we set the values of E=5 and D=5.

To analyze the network complexity, we take into consideration three encoder-decoder architectures in literature, i.e., SegNet [42] and InspectionNet [43]. Since the encoder and decoder networks in SegNet architecture follow the topology of the VGG-16 architecture, we computed the complexity of this network. The number of computations required for the ANet-FSM architecture is compared with the VGG-16 network architectures in Table 3.

The number of total computations performed by VGG-16 architecture is 38,016. Both the encoder and decoder network in SegNet follows VGG-16 architecture topology. Consequently, the number of convolution layers, kernels, and final computations increase by two times of the original VGG-16 architecture. The InspectionNet architecture performs 3806 fewer computations than SegNet. On the other hand, the ANet architecture performs 12,152 computations. Despite using a larger convolution kernel, this architecture performs 22,408 fewer computations than InspectionNet. Usage of a single convolution layer in each encoder along with less filter numbers reduced the computational complexity of ANet-FSM architecture. This aspect of the network helps eliminating the effect of gradient vanishing problem of deep networks. Furthermore, the precision of the network increases significantly due to this fact. Therefore, ANet-FSM architecture is the most in-expensive network in terms of computation in comparison to the networks represented in Table 3.

#### 3.2.2. Quantitative Comparisons

This section presents the performance of different deep architectures in the literature for concrete crack identification along with ANet-FSM architecture quantitatively. Deep concrete crack detection architectures are categorized as image classification-based architectures (Gibbs [3]) and encoder-decoder-based architectures (SegNet [42], InspectionNet [43], SegNet-SO [4]). We evaluated the performance of these networks using the test dataset of 200 images of size 1024×1024.

Statistical measures, shown in Table 4, such as true positive (TP) rate, false positive (FP) rate, true negative (TN) rate, false negative (FN) rate, error rate, and accuracy generate biased evaluation results toward non-crack pixels because of crack pixels low appearance. The use of effective statistical method, such as specificity, sensitivity, precision, recall, and F1 measure was also employed to distinguish between crack and non-crack pixels within imbalanced datasets. These statistical measures were identified by defining crack pixels as positive class and non-crack pixels as negative class. We define the TP rate as the percentage of accurately identified crack pixels, whereas the TN rate is correctly identified non-crack pixels. The FP rates are delineated as the percentage of wrongly identified crack pixels and FNs are the incorrect identification percentage of non-crack pixels. We measure how exactly a method can distinguish between the crack and non-crack pixels with the precision measure in an imbalanced dataset. We quantify the proportion of crack pixels identified accurately by a network with a recall score. The sensitivity measure evaluates the architecture’s responsiveness toward the aberrant behavior of defected pixels. Specificity measure is used to quantify the behavior of non-crack pixels. The overall performance of a network is evaluated using the F1 score. A summary of the quantitative measures used in this work is shown in Table 4. In addition, we assigned a ranking to the architectures based on both dependent and independent measures.

We evaluated the results of the proposed ANet-FSM architecture with three different thresholds. For applications in which high specificity and precision are required, higher threshold values should be utilized—see ANet-FSM (hi) in Table 5. Lower thresholds would be effective in applications that require higher sensitivity and recall score is needed—see ANet-FSM (low) in Table 5. In addition to these thresholds, we proposed the use of an optimal threshold, calculated using optimal thresholding algorithms such as the Otsu’s method [52], when no preference is given for performance measure with respect to the positive or the negative classes—see ANet-FSM (opt) in Table 5.

The overall results of different architecture and their average ranking on all the measures are shown in Table 6. In addition to this, each network architecture is assigned ranking on individual measures on Table 5, with 8 being the least accurate. Due to the block-based analysis technique for crack detection, [3] was ranked at 8, where lower rank corresponds to better performance of the algorithm and vice versa. This architecture classifies a sub-image of size 256×256 as crack or non-crack. Since crack pixels occur only a very small portion of an image, an enormous amount of pixels are falsely classified in these blocks. As a result, the false identification rate (both FP rate and FN rate), accuracy as well as the error rate of the Gibbs network is the worst among all the networks. This also represents the inefficiency of image classification methods in concrete crack identification and anomaly detection.

As expected, encoder-decoder architectures in Table 5 and Table 6 outperform classification networks such as the Gibbs architecture. Although SegNet [42] outperformed all the previous architectures for semantic segmentation in the field of scene parsing, the extremely imbalanced nature of concrete defect detection drops the performance of this architecture considerably. Additionally, the effect of the gradient vanishing problem (the result of an excessive number of layers) is reflected in evaluation measures such as FP and FN rates. The high false classification rate is also responsible for non-contributing feature maps generated due to the textured nature of the concrete surface. It is worth noting that these irrelevant feature maps are eliminated in ANet-FSM, as shown in better performance in all measures, compared to SegNet and shown in Table 6. For these reasons, SegNet architecture is not appropriate for solving the crack identification problem. As a result, SegNet achieves a low overall and individual ranking in all of the evaluation measures in Table 5 and Table 6.

The gradient vanishing problem affecting the SegNet was addressed by up-sampling the feature space of each encoder layer in Segnet-SO [4] architecture. This approach achieved higher TP rate and less error rate than SegNet architecture. Although the InspectionNet architecture improves the TP rate significantly, the highest FP rate in Table 6 demonstrates the effect of gradient vanishing problem. SegNet-SO architecture is more robust to the gradient vanishing problem despite achieving a lower TP rate than InspectionNet. Therefore, it is worth mentioning that, none of the architectures discussed above represent robustness in all the measures.

The ANet-FSM architectures addresses the drawbacks of SegNet, SegNet-SO, and InspectionNet architectures by eliminating redundant computation. These computationally expensive methods represent a fluctuation in results. For example, InspectionNet obtains outstanding correct classification with the cost of an unacceptable misclassification rate. SegNet-SO degrades the correct classification rate in the course of reducing incorrect classification. To evaluate the stability of the ANet-FSM architecture, we have analyzed its performance without incorporating the FSM (referred to as ANet). The robustness of ANet architecture is reflected by the ranking of 4 in each measure in Table 5. Although the InspectionNet architecture performs better in positive classification (TP), the low negative classification rate (TN) represents the unfeasible nature of this network towards imbalanced dataset. Therefore ANet architecture is substantially stable (the effect of using 7×7 spatial neighborhood in the convolution) despite achieving a lower ranking in some measures. However, the higher false positive rate of this network than InspectionNet represents that it is affected by the vanishing gradient problem because of a 7×7 kernel size. The association of the FSM model significantly addresses this problem as well as improves the performance in all measures.

To further investigate the result of feature silencing, we performed the thresholding operation on the result obtained from the ANet-FSM architecture. We discarded the crack pixels having a lower probability than a specific threshold in this operation. Three different threshold values were set experimentally to perform this operation such as high (15%), low (10%), and optimal (14%). It is evident from Table 5 and Table 6 that ANet-FSM(low) architecture achieves highest performance in all but four measures. Specifically, ANet-FSM (low) has recognized the highest number of crack pixels among all the networks in Table 5. However, the lowest TN and FP rates represent this network’s bias toward only positive classification. Therefore, this thresholding is suitable for application requiring to classify only crack locations. On the other hand, when a higher threshold is applied to ANet-FSM architecture, the FP rate significantly drops with the cost of a low TP rate. This thresholding technique is appropriate for applications that need to know healthy concrete locations. An optimal threshold was set experimentally to achieve a better TP rate and moderately lower FP rate than the previous networks. The ANet-FSM (opt) architecture outperforms all the aforesaid networks in every measure with a rank of 2. Although ANet-FSM(opt) does not perform best in all of the measures, the second-best ranking represents its stability in identifying both crack and non-crack pixels.

The above discussion evaluates the result of different architectures based on dependent evaluation measures. As mentioned earlier, these measures are highly biased toward the classification of the overwhelming majority class (non-crack). Nonetheless, to perform fair evaluation we have taken into account some measures such as precision, recall, sensitivity, specificity, and F1-score.

The Gibbs method [3] and SegNet [42] architecture have the lowest precision and recall score in Table 5 and Table 6. SegNet-SO architecture has a higher precision rate than InspectionNet architecture. However, the recall score represents a reverse relationship between SegNet-SO and InspectionNet. This tension between precision and recall is a well-known phenomenon within classification problems suffering from class-imbalance issues. Moreover, specificity and sensitivity represent similar relationships as precision and recall due to excessive class imbalance present in concrete crack datasets. If a network is highly specific, its sensitivity reduces (SegNet-SO) whereas a high sensitivity rate reduces the specificity of a network (InspectionNet). As a result, the F1 score is widely used to combine the effects of these measures for any machine learning architecture. The F1 score of SegNet-SO and InspectionNet represents that the former is better in terms of overall performance.

On the other hand, the ANet architecture maintains stable precision, recall, specificity, and sensitivity scores (all are assigned the same rank in Table 5). Consequently, its F1 score is better than SegNet-SO and less than InspectionNet because of lower sensitivity. However, the ANet-FSM architecture with a low threshold achieves exceptional recall scores with relatively low specificity, resulting in the highest F1 score of all the methods. If extreme thresholding is applied, ANet-FSM architecture obtains the highest precision and specificity score with moderately low recall and sensitivity score among all the architectures. The optimal thresholding operation achieves higher precision, recall, specificity, sensitivity, and F1 scores than all the networks in Table 5 and Table 6. The same ranking in all of the measures in Table 5 also demonstrates the stability of the network. This architecture obtains the highest F1-score among all the computationally expensive networks (SegNet, InspectionNet, SegNet-SO). Therefore, it can be concluded that this network is appropriate for use in applications with highly class-imbalanced data.

It is worth mentioning the rationale behind the need for achieving high degrees of accuracy for concrete crack detection compared to generic semantic segmentation applications. In generic semantic segmentation, very high accuracy values (both true positive and true negative rates) might indicate overfitting. Overfitting causes the network to have very low loss values and high accuracy values on training samples by the accuracy will drop on the test samples. In crack detection applications, due to the significant imbalanced nature of the dataset, i.e., significantly fewer crack pixels compared to non-crack pixels, maintaining higher generalization rates becomes important. As it can be seen from Table 6, generic segmentation networks such as SegNet and InspectionNet might be suffering from overfitting. This can be observed by very high true negative values (98.9%, 99.1%, 98.8%, and 99.0% for SegNet, SegNet-SO, InspectionNet, and ANet, respectively) but a significantly lower true positive rates (72%, 73%, 81%, and 75% for SegNet, SegNet-SO, InspectionNet, and ANet, respectively). However, ANet-FSM${}_{opt}$ addresses this problem by establishing irrelevant feature maps to crack detection (which suffer from the imbalance in the number of crack and non-crack pixel), while finding an optimal threshold value for the likelihood of a pixel belonging to the distribution of crack pixels vs. non-crack pixels. This fact can be observed by high true negative and true positive values. Moreover, the goal of the network is the detection of crack pixels, and the achieving 87% true positive rate shows that the network has learning discriminating features with high levels of accuracy while avoiding the overfitting problem.

To perform an unbiased comparison, we have evaluated the performance of the proposed architecture using the evaluation metrics from Berkeley segmentation benchmark [53]. Three evaluation metrics from the benchmark were employed to assess the performance of the networks such as boundary displacement error (BDE), global consistency error (GCE), and variation in information (VI). The BDE measures the distance between the boundary pixels between two segmented images. GCE represents how closely two segmented images can be shown as a representation of one another. The VI is used widely for data clustering applications. It measures the distance between two clusters (resembles mutual information). Since the probabilistic rand index replicates the same measurement as the accuracy of the algorithms, we have avoided this measurement. For comparison purposes, we have considered the SegNet, InspectionNet, and the proposed ANet-FSM architecture. The SegNet architecture was chosen by us to evaluate the effect of gradient vanishing problem on state-of-the-art semantic segmentation network. We have chosen InspectionNet to evaluate the effect of gradient vanishing problem in crack detection architecture. The comparison of different methods on these metrics on different datasets are shown in Table 7.

We used three different types of datasets for performing a fair evaluation of the proposed method. These metrics were applied on Crack260 [44], CrackForest [50] and Illinois Bridge dataset. The Crack260 and CrackForest datasets are published annotated datasets for crack classification. The Illinois Bridge dataset was collected and prepared by the researchers of Advanced Robotics and Automation Lab. In Table 7, for the Crack260 dataset, SegNet achieves the lowest BDE, whereas InspectionNet achieves the highest BDE. Considering the sheer number of parameters involved in SegNet, this low error rate is reasonable. In InspectionNet the number of parameters is more than SegNet. Due to the parameter degradation problem, the BDE is highest in this network. The ANet-FSM architecture achieves a BDE close to SegNet, despite having almost half the number of parameters as SegNet. On the other hand, for CrackForest and Illinois Bridge datasets, the BDE is lowest in ANet-FSM architecture. This represents that pruning feature space significantly enhances network performance. For Illinois dataset, the BDE of ANet-FSM is seven times lower than InspectionNet and twelve times lower than SegNet. For CrackForest dataset, the BDE of ANet-FSM is 1.8 times lower than InspectionNet and 2.5 times lower than SegNet. These two datasets significantly represents the effect gradient vanishing problem in complex architectures like SegNet and InspectionNet.

ANet-FSM architecture achieves the lowest GCE in CrackForest and Illinois Bridge datasets. However, for the Crack260 dataset, it achieves the second-best result among all the methods. On the other hand, ANet-FSM architecture achieves best VI for the Illinois Bridge dataset. For CrackForest dataset, InspectionNet achieves the best VI. SegNet achieves the best VI for the Crack260 dataset.

The rand index metric in the Berkeley segmentation benchmark represents the previously analyzed measure accuracy. Since, accuracy is dependent on TP, FP, FN, and TN for measurement, it was not incorporated for evaluation in Table 7 and Table 8. In addition, the region uniformity measure is widely used in semantic segmentation architecture evaluation. Region uniformity is more appropriate for image segmentation problems such as scene parsing and medical image analysis. These segmentation problems identify a region containing a substantial amount of pixels. Unlike these regions, crack width length can be of one pixel to several pixels. The crack area encompasses a very small number of pixels (usually five to ten pixels approximately). For this reason, VI and GCE measures are not directly applicable to the crack segmentation problem also. As a result, the GCE present in Table 7 is higher than usual and the VI measure shows different behavior for each individual dataset.

We have also compared the performance of ANet-FSM architecture with state-of-the-art crack detection architectures such as DeepCrack [44] and SDDNet [45]. The results of deep crack and SDDNet were extracted from the experiments reported in [44]. The ANet-FSM architecture was trained and tested using the dataset [44] used for the experiment in [45]. For comparison metrics, we have taken into consideration the mean intersection over union (mIou) [45], precision, recall, F1 score, and the processing time. The results are shown in Table 8. SDDNet architecture achieves the highest F1 score and mIou among all the methods. However, the ANet-FSM architecture achieves the highest precision rate and an F1 score close to SDDNet. This phenomenon represents the effect of gradient vanishing in lowest in ANet-FSM among all the methods. Moreover, the processing time of ANet-FSM is 1.14 ms per image, whereas SDDNet has 13.04 ms per image. The processing speed of ANet-FSM architecture is thirteen-time smaller than the SDDNet architecture. Considering this significant fast processing time of ANet-FSM architecture, the smaller mIou score is reasonable. Additionally, this processing time also depicts ANet-FSM architecture is nominally affected by gradient stability problem.

#### 3.2.3. Qualitative Comparisons

**Figure 10:**
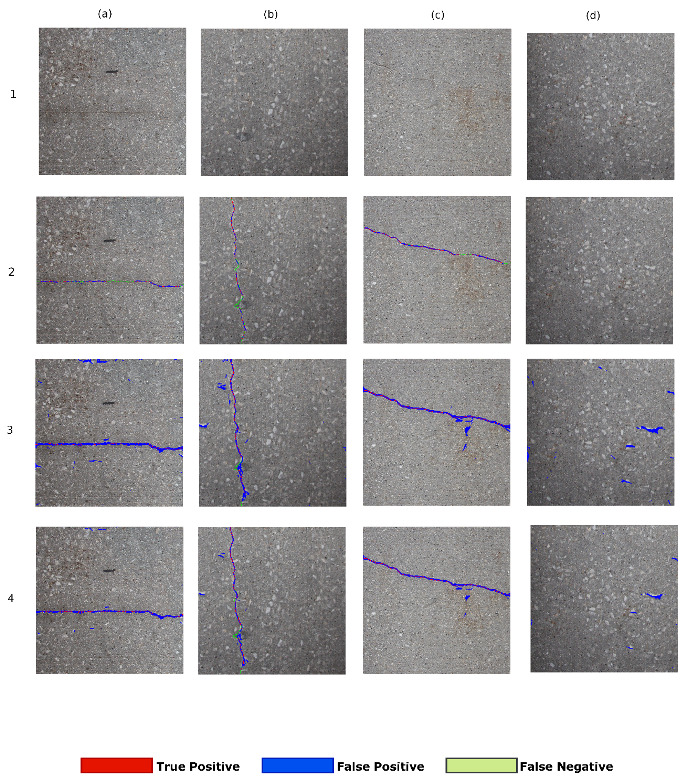
Comparison of different thresholds on the performance of ANet-FSM on four sample images. Columns are the tested images: (**a**) contains clear horizontal crack, (**b**) contains clear vertical crack, (**c**) contains a crack at arbitrary orientation, (**d**) non-cracked concrete image. Row 1—original image; Row 2—ANet-FSM (hi); Row 3—ANet-FSM (low); and Row 4—ANet-FSM (opt). Results will be seen clearer when zoomed in.

The qualitative comparison of several crack identification networks is performed in this section. We first show the results of ANet-FSM architecture in Figure 10. Then we compare our results with the image classification method (Gibbs [3]). Finally, the results are compared with the state-of-the-art encoder-decoder architectures such as SegNet [42], SegNet-SO [4], and InspectionNet [43]. We color-coded the true positive pixels (correctly detected crack pixels) with red, FPs (missed crack pixels) with blue, and FNs (pixels incorrectly labeled as crack) with green, respectively. The TN pixels (correctly labeled non-crack) are represented with their original texture.

We have represented the results of ANet-FSM architecture on three thresholding values such as high (15%), low(10%), and optimal (14%) in Figure 10. If a low threshold is applied, the network becomes more sensitive towards the environmental non-uniformity and gradient vanishing problem. On the other hand the network becomes more specific to correct classification. As a result, the number of falsely identified (blue colors) and correctly classified pixels (red colors) of the network significantly increases (shown in Figure 10(2)a–c). When a high threshold is applied (shown in Figure 10(3)a–c) the sensitivity towards noise is resolved but the specificity of correct classification also decreases. Application of an optimal threshold not only reduces the false classification rate but also increases correct classifications significantly. This thresholding obtains a balance between specificity and sensitivity as shown in Figure 10.

**Figure 11:**
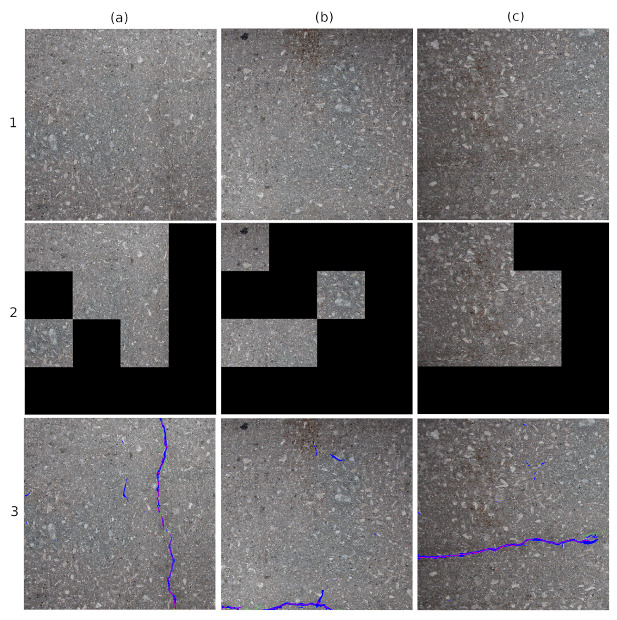
Comparison of image classification architectures with encoder-decoder architecture on three sample images. Columns are the tested images: (**a**) contains clear vertical crack, (**b**) contains small crack on bottom, (**c**) contains a crack at arbitrary orientation. Row 1—original image; Row 2—Gibbs architecture [3]; Row 3—ANet-FSM architecture.

We compared the results of the Gibbs [3] architecture with ANet-FSM architecture in Figure 11. Gibbs architecture divides the original image into smaller sub-blocks of size 256×256 and classifies them as crack and non-crack. The non-crack blocks are marked as black pixels in Figure 11 and crack blocks are represented with their original texture. The results represent that, Gibbs architecture falsely identifies many crack blocks as well as fails to identify the exact location of cracks. On the other hand, the ANet-FSM architecture localize and identify crack location more precisely than the Gibbs architecture. For example, in Figure 11(2)a the Gibbs method falsely identifies four 256×256 blocks as crack blocks. The ANet-FSM architecture in Figure 11(3)a represents the exact crack location as well as misidentifies a very less number of crack pixels in comparison to Gibbs architecture.

**Figure 12:**
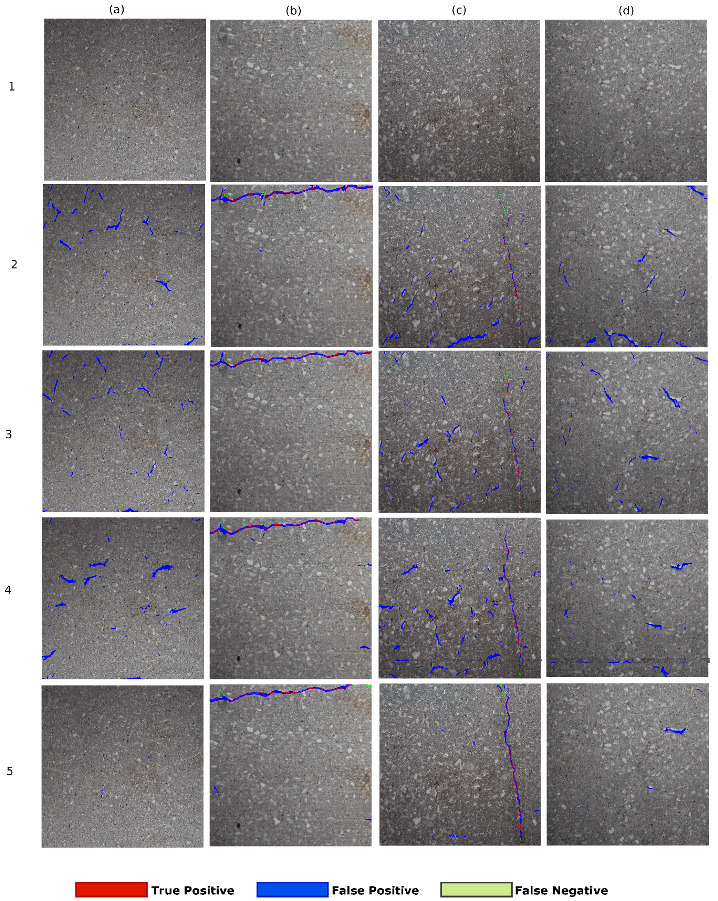
Comparison of different encoder-decoder-based architectures on four sample images. Columns are the tested images: (**a**) contains non-cracked concrete image, (**b**) contains clear horizontal crack on top, (**c**) contains clear vertical crack, (**d**) contains non-crack concrete image. Row 1—original image; Row 2—SegNet [42]; Row 3—SegNet-SO [4]; Row 4—InspectionNet; Row 5—ANet-FSM architecture.

The result of different encoder-decoder networks such as SegNet, SegNet-SO, and InspectionNet and the proposed ANet-FSM architecture is shown in Figure 12.

The results in Figure 12(2)a,c,d show that the SegNet architecture has more falsely classified pixels (blue colored) than the remaining networks. The excessive number of feature space (due to maximal network complexity), as well as the vanishing gradients, contribute to this false classification. The SegNet-SO architecture in Figure 12(3)b moderately removes the false classification present in Figure 12(2)b. The results in Figure 12(3)a,c,d represent an considerable amount of falsely classified pixels, specifically in Figure 12(3)a where no crack pixels are present originally. On the other hand, the InspectionNet architecture represented in Figure 12(4)a–d shows a performance improvement in comparison to SegNet and SegNet-SO. The results in Figure 12(4)a,d are less affected by the FP rate than SegNet and SegNet-SO. However, the false identification rate increases more than SegNet-SO architecture when a significant number of crack pixels are present as shown in Figure 12(3)c. As a result, it can be interpreted that InspectionNet is highly unstable as well as affected by the environmental non-uniformity (lighting and shading). On the other hand, the effect of FPs is significantly low in ANet-FSM architecture in comparison to the results in row-2, row-3, and row-4. It has almost no false identification (blue pixels) in Figure 12(5)a. There exists a small amount of falsely identified pixels in Figure 12(5)d, which is considerably lower than the previous networks. Moreover, this network improves the false classification without affecting the correct classification rate (represented as red pixels in Figure 12(5)b,c), which is the effect of feature silencing. The ANet-FSM architecture not only improves the accuracy of crack identification but also eliminates the effect of false identification significantly with the FSM. Therefore, it can be concluded, ANet-FSM architecture is less effected by the gradient vanishing problem in comparison to the encoder-decoder architectures in [4,42,43]. Based on the percentage of correctly identified crack pixels, it can be concluded that ANet-FSM provides performance that is an improvement on the state-of-the-art encoder decoder network architectures designed for crack detection in the recent past.

**Figure 13:**
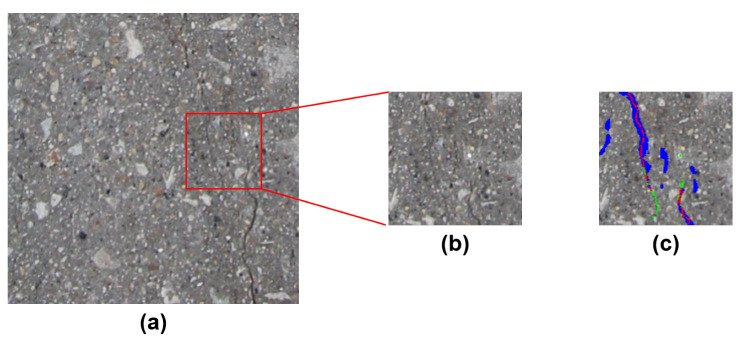
ANet-FSM detection results on images containing very small and vague crack paths. (**a**) The original image. (**b**) The magnification of the area with a vague crack path. (**c**) The detection results of ANet-FSM.

Figure 13 showcases the robustness and accuracy of the proposed ANet-FSM architecture in detecting very small cracks on concrete surfaces. The image shown in Figure 13a contains a crack that passes vertically through the surface, with the middle portion (the red square) being thin and almost not discernible. Figure 13b shows a magnification of this area. The detection results are shown in Figure 13c.

## 4. Conclusions and Future Work

A deep convolutional neural network for concrete crack classification is presented in this work. A framework for eliminating the effect of gradient vanishing problem for class imbalanced dataset is presented in this work. Silencing unnecessary feature space enhances the precision of our framework as well as reduces the effect of the gradient vanishing problem. Moreover, the sensitivity of the architecture to environmental non-uniformity is reduced by the FSM module. As a result, our architecture is more precise than the crack detection methods present in the literature. The experimental results in this study also represent that an enormous amount of unnecessary computations is performed in deep architectures. Elimination of these computations enhance the speed and processing of deep networks remarkably, which is important for real-time deployment of this architectures. The FSM module selects features based on some threshold in the proposed framework. In the future, we want to develop an optimization framework for feature silencing in class imbalanced datasets. Another future direction of this work would be exploring the area of other concrete distress identification such as spalling detection using an optimized feature silencing module.

## Figures and Tables

**Table 1:** Literature review of approaches for concrete crack detection and their advantages and shortcomings.

	Image Processing Based				Machine Learning Based				Deep Learning Based	
Comparison	Threshold	Morphology	Edge		SVM	Adaboost	MLP		Classifier	Segmentation
Cost	Low	Low	Low		Low	Med	Med		High	High
Memory	Low	Low	Low		Low	Med	Med		High	High
Accuracy	Low	Low	Low		High	Med	Med		Low	High
Robustness	Low	Low	Low		Med	Med	Med		High	High
Generalization	Low	Med	Low		High	High	High		High	High

**Table 2:** FSM from an instance of the Illinois Bridge dataset. Once the image is passed through multiple encoders, the image size is reduced to half of the original. †: Feature silencing rate.

Encoder Kernel Size	FSR${}^{†}$		Feature Space Size			No. Features $F_{s}$			No. of Silenced Features
			Before	After		Before	After		
256×256	00.0%		262,144	262,144		4	4		0
128×128	50.0%		132,072	65,536		8	4		4
64×64	43.8%		65,536	36,864		16	9		7
32×32	50.0%		32,768	16,384		32	16		16
16×16	53.1%		16,384	7680		64	30		34
Total Encoder Module	51.8%		508,904	388,608		124	63		

**Table 3:** Comparison of network complexity of different encoder-decoder-based architectures.

Method	No. of Conv. Layers	No. of Kernels	Kernel Size	Net. Complexity
VGG-16 [37]	13	4224	3×3	38,016
SegNet [42]	26	8064	3×3	72,576
InsepcionNet [43]	10	3840	3×3	34,560
ANet-FSM (ours)	5	248	7×7	12,152

**Table 4:** Quantitative measures used for evaluating the results of deep network architectures. Crack pixels belong to the positive class and non-crack pixels belong to the negative class.

Measure	Definition	Description
TP	True Positive	Number of accurately identified crack pixels
FP	False Positive	Number of pixels erroneously labeled as crack pixels
TN	True Negative	Number of accurately identified non-crack pixels
FN	False Negative	Number of crack pixels missed
Acc	Accuracy	(TP+TN)/(TotalPixels)
Err	Error Rate	(FP+FN)/(TotalPixels)
Spc	Specificity	TN/(TN+FP)
Sen	Sensitivity	TP/(TP+FN)
Prc	Precision	TP/(TP+FP)
Rec	Recall	TP/(TP+FN)
F1	F1-Score	(2×Pre×Rec)/(Pre+Rec)

**Table 5:** Ranking of each method based on individual quantitative measures.

Method	TP	FN	TN	FN	Acc	Err	Spc	Sen	Prc	Rec	F1
Gibb [3]	8	8	8	8	8	8	8	8	8	8	8
SegNet [42]	7	7	5	5	7	7	5	7	7	7	7
SegNet-SO [44]	5	5	3	3	5	5	3	5	3	5	6
InspectionNet [43]	3	3	6	6	3	3	6	3	6	3	3
ANet [4]	4	4	4	4	4	4	4	4	4	4	4
ANet-FSM${}_{hi}$ (ours)	6	6	1	1	6	6	1	6	1	6	5
ANet-FSM${}_{lo}$ (ours)	1	1	7	7	1	1	7	1	5	1	1
ANet-FSM${}_{opt}$ (ours)	2	2	2	2	2	2	2	2	2	2	2

**Table 6:** Overall ranking of each method based on quantitative measures.

			Dependent Measures							Indepndent Measures				
Method	Rank		TP	FN	TN	FN	Acc	Err		Spc	Sen	Prc	Rec	F1
Gibb [3]	8.0		25	75	77.0	23.0	51	49		77.0	25.2	52.3	50.7	51.5
SegNet [42]	6.5		72	28	98.9	1.1	85	15		98.9	71.9	98.5	77.9	87.0
SegNet-SO [4]	4.4		73	27	99.1	0.9	86	14		99.1	73.0	98.8	78.6	87.5
InspectionNet [43]	4.1		81	19	98.8	1.2	90	10		98.8	80.5	98.5	83.5	90.4
ANet (ours)	4.0		75	25	99.0	1.0	87	13		99.0	75.1	98.7	79.9	88.3
ANet-FSM${}_{hi}$ (ours)	4.1		72	28	**99.7** ${}^{†}$	**0.3** ${}^{†}$	86	14		**99.7** ${}^{†}$	72.2	**99.6** ${}^{†}$	78.2	87.6
ANet-FSM${}_{lo}$ (ours)	3.0 ${}^{‡}$		**90** ${}^{†}$	**10** ${}^{†}$	98.7	1.3	**94** ${}^{†}$	**6** ${}^{†}$		98.7	**89.8** ${}^{†}$	98.6	**90.6** ${}^{†}$	**94.3** ${}^{†}$
ANet-FSM${}_{opt}$ (ours)	**2.0** ${}^{†}$		87 ${}^{‡}$	13 ${}^{‡}$	99.2 ${}^{‡}$	0.8 ${}^{‡}$	93 ${}^{‡}$	7 ${}^{‡}$		99.2 ${}^{‡}$	86.6 ${}^{‡}$	99.1 ${}^{‡}$	88.1 ${}^{‡}$	93.3 ${}^{‡}$
	†: Best Method.					‡: Second Best Method.								

**Table 7:** Performance of crack identification in different dataset. The measures represented in this table are from Berkeley segmentation benchmark [53]. The lowest value of each measure represents the best.

Dataset	Method	BDE	GCE	VI
**Illinois Bridge**	ANet-FSM (ours)	**0.19**	**0.988**	**0.36**
	SegNet [42]	2.39	0.993	1.40
	InspectionNet [43]	1.42	0.995	0.39
**Crack260** [44]	ANet-FSM (ours)	1.84	0.992	1.53
	SegNet [42]	**1.72**	**0.991**	**0.92**
	InspectionNet [43]	2.40	0.992	1.01
**CrackForest** [50]	ANet-FSM (ours)	**0.89**	**0.992**	1.22
	SegNet [42]	2.20	**0.992**	0.79
	InspectionNet [43]	1.60	0.980	**0.78**

**Table 8:** Comparison of ANet-FSM architecture with crack detection architectures. The ANet-FSM architecture was trained and tested on the dataset prepared by DeepCrack [44].

Method	Prc	Rec	F1	mIOU	Processing Time
DeepCrack [44]	86.1%	86.9%	86.5%	80.2%	109 ms
SDDNet [45]	87.1%	**87.0**%	87.0%	**87.9**%	13.54 ms
ANet-FSM (ours)	**98.7**%	79.9%	**88.3**%	79.0%	**1.14** ms

## Abbreviations

The following abbreviations are used in this manuscript:

CNN	Convolutional Neural Networks
FSM	Feature Silencing Module
ANet	Attention Network
DoG	Difference of Gaussians
ANN	Artificial Neural Networks
SVM	Support Vector Machines
MLP	Multi Layer Perceptron
BN	Batch Normalization
ReLU	Rectified Linear Unit
TP	True Positive
FP	False Positive
TN	True Negative
FN	False Negative
SO	Side Outputs
BDE	Boundary Displacement Error
VI	Variation in Information
GCE	Global Consistency Error
